# Dynamic driving pressure associated mortality in acute respiratory distress syndrome with extracorporeal membrane oxygenation

**DOI:** 10.1186/s13613-017-0236-y

**Published:** 2017-01-25

**Authors:** Li-Chung Chiu, Han-Chung Hu, Chen-Yiu Hung, Chih-Hao Chang, Feng-Chun Tsai, Cheng-Ta Yang, Chung-Chi Huang, Huang-Pin Wu, Kuo-Chin Kao

**Affiliations:** 1 Department of Thoracic Medicine, Chang Gung Memorial Hospital, Chang Gung University College of Medicine, Linkou, No. 5, Fu-Shing St., Kwei-Shan, Taoyuan, 886 Taiwan; 2 Department of Respiratory Therapy, Chang Gung Memorial Hospital, Chang Gung University College of Medicine, Taoyuan, Taiwan; 3 Department of Respiratory Therapy, Chang Gung University College of Medicine, Taoyuan, Taiwan; 4 Division of Cardiovascular Surgery, Chang Gung Memorial Hospital, Taoyuan, Taiwan; 5 Division of Pulmonary, Critical Care and Sleep Medicine, Chang Gung Memorial Hospital, Keelung, Taiwan

**Keywords:** Driving pressure, Mechanical ventilation, Acute respiratory distress syndrome, Extracorporeal membrane oxygenation, Outcome

## Abstract

**Background:**

The survival predictors and optimal mechanical ventilator settings in patients with severe acute respiratory distress syndrome (ARDS) undergoing extracorporeal membrane oxygenation (ECMO) are uncertain. This study was designed to investigate the influences of clinical variables and mechanical ventilation settings on the outcomes for severe ARDS patients receiving ECMO.

**Methods:**

We reviewed severe ARDS patients who received ECMO due to refractory hypoxemia from May 2006 to October 2015. Serial mechanical ventilator settings before and after ECMO and factors associated with survival were analyzed.

**Results:**

A total of 158 severe ARDS patients received ECMO were finally analyzed. Overall intensive care unit (ICU) mortality was 55.1%. After ECMO initiation, tidal volume, peak inspiratory pressure and dynamic driving pressure were decreased, while positive end-expiratory pressure levels were relative maintained. After ECMO initiation, nonsurvivors had significantly higher dynamic driving pressure until day 7 than survivors. Cox proportional hazards regression model revealed that immunocompromised [hazard ratio 1.957; 95% confidence interval (CI) 1.216–3.147; *p* = 0.006], Acute Physiology and Chronic Health Evaluation (APACHE) II score (hazard ratio 1.039; 95% CI 1.005–1.073; *p* = 0.023), ARDS duration before ECMO (hazard ratio 1.002; 95% CI 1.000–1.003; *p* = 0.029) and mean dynamic driving pressure from day 1 to 3 on ECMO (hazard ratio 1.070; 95% CI 1.026–1.116; *p* = 0.002) were independently associated with ICU mortality.

**Conclusions:**

For severe ARDS patients receiving ECMO, immunocompromised status, APACHE II score and the duration of ARDS before ECMO initiation were significantly associated with ICU survival. Higher dynamic driving pressure during first 3 days of ECMO support was also independently associated with increased ICU mortality.

## Background

Acute respiratory distress syndrome (ARDS) is a heterogeneous syndrome with complex pathophysiologic mechanisms and has a high mortality rate up to 45% in severe ARDS [1]. A lung-protective ventilation strategy with lower tidal volume remains the cornerstone of treatment for ARDS and is associated with improved survival [2]. Many alternative rescue treatments had been investigated for ARDS with severe hypoxemia, but their impact on mortality is undetermined, except for early application of prolonged prone position [3].

Although the survival benefit is not well established, extracorporeal membrane oxygenation (ECMO) may be a salvage therapy for severe ARDS patients with profound hypoxemia refractory to conventional mechanical ventilation [4–6]. For severe ARDS patients receiving ECMO support, the positive result of a multicenter randomized controlled trial [7], favorable outcomes during the 2009 influenza A (H1N1) pandemic [8] and major advances in technology with less complication had allowed ECMO widespread application over the past decade [5, 9, 10]. However, the precise indications, optimal timing to initiate and factors associated with mortality for severe ARDS patients who received ECMO were still not well established [4, 6, 9, 11].

ECMO facilitates an ultra-protective ventilation of more lowering delivered tidal volume and airway pressure for resting the lungs. This ultra-protective lung strategy ideally may improve outcomes by further minimizing ventilator-induced lung injury (VILI) [4, 6, 11–16]. Although ECMO support limited stress and strain with ultra-protective ventilation, the specific extent of lung rest strategy and the optimal mechanical ventilation settings targets during ECMO for severe ARDS patients remained uncertain [11–16]. There was no large multicenter prospective randomized controlled trial to address the optimal mechanical ventilation settings during ECMO in severe ARDS patients. In most clinical practice, the mechanical ventilation settings during the ECMO depended on the clinicians’ experience [6]. A recent study from 3562 patients with ARDS enrolled in 9 previous reported randomized controlled trials concluded that decreases in driving pressure were strongly associated with increased survival for patients with ARDS [17]. However, it is uncertain whether a similar association between driving pressure and survival exists for severe ARDS patients receiving ECMO.

The aim of this study was to investigate the influences of clinical variables and mechanical ventilation settings on the survival outcomes for severe ARDS patients receiving ECMO.

## Methods

### Patient papulation

This study was conducted in the medical and surgical ICUs at a tertiary care referral center, Chang Gung Memorial Hospital, with a 3700-bed general ward and a 278-bed adult ICU. The local Institutional Review Board for Human Research approved this study (CGMH IRB No. 201600632B0), and the need for informed consent was waived due to the retrospective nature of the study.

We analyzed severe ARDS patients who received ECMO for refractory hypoxemia between May 2006 and October 2015. Severe ARDS was defined by the Berlin definition with acute onset within 1 week, bilateral lungs opacities, no evidence of cardiac failure-related hydrostatic edema by echocardiography and PaO_2_/FiO_2_ ratio <100 mmHg with positive end-expiratory pressure (PEEP) ≥5 cm H_2_O [1]. Exclusion criteria were: (1) age <20 years, (2) malignancies with poor prognosis within 5 years and (3) significant underlying comorbidities or severe multiple organ failure refractory to treatment (4) mortality within 24 h after ECMO initiation. Before consideration of ECMO initiation, all patients were sedated and ventilated with pressure-controlled ventilation using tidal volume of 6–8 ml/kg predicted body weight (PBW).

### ECMO management and protocol

The decision to initiate ECMO was made by treating intensive care specialist when persistent hypoxemia (PaO_2_/FiO_2_ ratio <80 mmHg) at least 6 h, despite aggressive mechanical ventilation support (PEEP > 10 cm H_2_O or peak inspiratory pressure >35 cm H_2_O). All patients were deeply sedated and paralyzed with continuous neuromuscular blocking agent and ventilated with pressure-controlled ventilation until weaning attempt from ECMO. Initial mechanical ventilator settings protocol after ECMO support were as follows: tidal volume 4–6 ml/kg PBW; PEEP 10–15 cm H_2_O; peak inspiratory pressure 25–30 cm H_2_O; respiratory rate 10–12 breaths per minute; and FiO_2_ adjusted to maintain arterial oxygen saturation above 90%. The criteria for weaning from ECMO in our experience were resolving lungs infiltration, lung compliance >20 ml/cm H_2_O, PaO_2_ > 60 mmHg and PaCO_2_ < 45 mmHg under FiO_2_ ≦ 0.4, PEEP ≦ 6–8 cm H_2_O, and peak inspiratory pressure ≦30 cm H_2_O.

### Data collection

The following data were collected from the hospital chart and analyzed: age, sex, body weight, body mass index, etiologies of ARDS, underlying diseases, Acute Physiology and Chronic Health Evaluation (APACHE) II score, Sequential Organ Failure Assessment (SOFA) score and lung injury score on the day of ICU admission.

Arterial blood gas, ARDS duration before ECMO, ventilator settings included tidal volume, respiratory rate, PEEP, peak inspiratory pressure, dynamic driving pressure (the difference between peak inspiratory pressure and PEEP) and FiO_2_ were recorded before ECMO initiation. After ECMO support, daily arterial blood gas, ventilator settings, ECMO settings (gas flow, blood flow and FiO_2_) and ECMO complications (oxygenator failure, blood clots in oxygenator or circuit, bleeding, infection or others) were recorded until ICU discharge.

### Statistical analyses

Continuous variables were presented as means ± standard deviation or median (interquartile range), and categorical variables were reported as numbers (percentages). Student’s *t* test or the Mann–Whitney *U* test was used to compare continuous variables between survivors and nonsurvivors, as appropriate. Categorical variables were tested using Chi-square test for equal proportion or Fisher’s exact test. Risk factors associated with ICU mortality were analyzed using Cox proportional hazards regression model with stepwise selection procedure. All variables that were related to ICU mortality with a *p* < 0.20 were finally introduced in the model. Calibration was assessed using Hosmer–Lemeshow goodness-of-fit test (C statistic, goodness of fit was defined as a *p* value >0.05), and discrimination was assessed by the area under the receiver operating characteristics curve. Cutoff points were calculated by obtaining the best Youden index (sensitivity + specificity − 1). The results were presented as hazard ratio (HR) [95% confidence interval (CI)]. Cumulative survival curves as a function of time were generated using the Kaplan–Meier approach and compared using the log-rank test. All statistical analyses were performed with SPSS 21.0 statistical software. A *p* value <0.05 was considered significant.

## Results

**Fig. 1:**
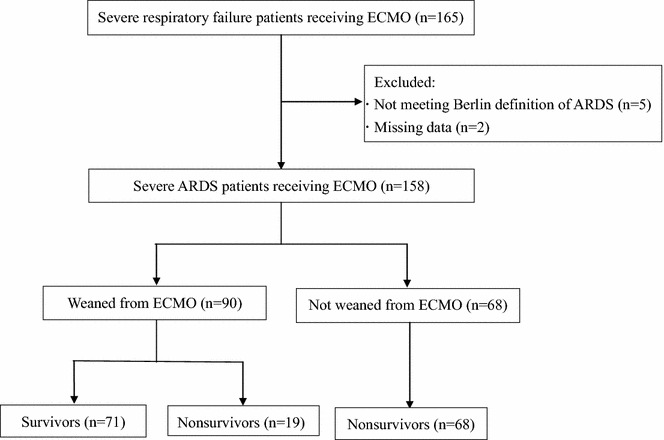
Flowchart of severe acute respiratory distress syndrome (ARDS) patients receiving extracorporeal membrane oxygenation (ECMO)

During the study period, 2622 patients were admitted to our ICUs with a diagnosis of ARDS, of whom 165 patients with severe ARDS receiving ECMO were included. A total of 158 patients were finally analyzed (Fig. 1). The overall ICU survival rate was 44.9%.

Details of the demographic data, clinical characteristics and ventilator settings before ECMO initiation between survivors and nonsurvivors are presented in Table 1. The main cause of ARDS was bacterial pneumonia, followed by viral pneumonia. Survivors were younger and had less immunocompromised and lower baseline APACHE II, SOFA scores than nonsurvivors. The duration of ARDS before ECMO initiation was significantly shorter in survivors than nonsurvivors. Mechanical ventilation settings and other ventilation parameters before ECMO support in two groups did not show significantly difference. Venovenous ECMO was used for 120 patients (75.9%), and other 38 patients received venoarterial ECMO with 11 patients shifted to venovenous ECMO later. Venoarterial ECMO was performed for heart failure with intractable shock complicating ARDS-related diseases. The median duration of ECMO, ventilator, ICU and hospital stay were 9 (5–15), 20 (12–38), 23 (13–43) and 39 (21–64) days, respectively. Overall, 43 (27.2%) patients had one or more ECMO-related complications with 4 patients died due to intracranial hemorrhage.

**Table 1:** Characteristics of survivors and nonsurvivors of severe ARDS patients with ECMO support

Characteristic	All patients (*n* = 158)	Survivors (*n* = 71)	Nonsurvivors (*n* = 87)	*p* value
Age (years)	50.3 ± 16.3	46.0 ± 16.5	53.8 ± 15.4	0.003
Male (gender)	108 (68.4%)	48 (67.6%)	60 (69%)	0.855
Body weight (kg)	68.5 ± 16.7	70.1 ± 17.4	67.1 ± 16.1	0.268
Body mass index (kg/m^2^)	25.8 ± 5.2	26.0 ± 5.8	25.6 ± 4.7	0.656
ARDS etiologies				
Bacterial pneumonia	55 (34.8%)	19 (26.8%)	36 (41.4%)	0.055
Viral pneumonia	24 (15.2%)	13 (18.3%)	11(12.6%)	0.327
Nonpulmonary sepsis	21 (13.3%)	4 (5.6%)	17 (19.5%)	0.017
Pulmonary contusion	19 (12%)	13 (18.3%)	6 (6.9%)	0.028
Aspiration pneumonia	11 (7%)	8 (11.3%)	3 (3.4%)	0.066
Other causes	28 (17.7%)	14 (19.7%)	14 (16.1%)	1.0
Comorbidities				
Diabetes mellitus	40 (25.3%)	23 (32.4%)	17 (19.5%)	0.065
Cerebrovascular accident	10 (6.3%)	6 (8.5%)	4 (4.6%)	0.346
Chronic heart disease^a^	55 (34.8%)	23 (32.4%)	32 (36.8%)	0.565
Chronic lung disease^b^	16 (10.1%)	4 (5.6%)	12 (13.8%)	0.115
Chronic liver disease^c^	22 (13.9%)	6 (8.5%)	16 (18.4%)	0.073
Chronic kidney disease^d^	18 (11.4%)	8 (11.3%)	10 (11.5%)	0.964
Immunocompromised^e^	42 (26.6%)	11 (15.5%)	31 (35.6%)	0.004
APACHE II score	23.4 ± 7.5	21.8 ± 8.0	24.7 ± 6.9	0.014
SOFA score	10.9 ± 3.2	10.3 ± 3.1	11.4 ± 3.2	0.042
Lung injury score	3.37 ± 0.44	3.44 ± 0.43	3.32 ± 0.45	0.085
ARDS duration before ECMO (h)	28.0 (7.0–129.0)	9.8 (3.7–64.0)	54.0 (16.0–200.0)	<0.001
Pre-ECMO ventilator settings				
PaO_2_/FiO_2_ (mmHg)	64 (52–87)	64 (53–80)	63 (52–107)	0.198
Tidal volume (ml/kg PBW)	7.7 ± 2.4	7.7 ± 2.3	7.8 ± 2.4	0.753
PEEP (cm H_2_O)	12.0 ± 2.8	12.2 ± 2.5	11.8 ± 3.0	0.319
Peak inspiratory pressure (cm H_2_O)	33.9 ± 6.5	33.6 ± 6.0	34.1 ± 6.8	0.645
Mean airway pressure (cm H_2_O)	18.7 ± 4.4	18.4 ± 4.2	18.9 ± 4.6	0.539
Dynamic driving pressure (cm H_2_O)	21.9 ± 6.2	21.1 ± 5.8	22.6 ± 6.5	0.139
Dynamic compliance (ml/cm H_2_O)	22.5 ± 11.2	23.4 ± 11.6	21.7 ± 10.9	0.366
Pre-ECMO blood gas				
pH	7.28 ± 0.14	7.27 ± 0.12	7.28 ± 0.15	0.842
PaCO_2_ (mmHg)	52.2 ± 18.8	50.7 ± 19.6	53.5 ± 18.2	0.359
PaO_2_ (mmHg)	73.3 ± 39.3	71.6 ± 39.2	74.8 ± 39.6	0.617
Saturation (%)	84.4 ± 15.9	84.7 ± 12.7	84.1 ± 18.2	0.804
Ventilator settings from day 1 to 3 on ECMO				
PaO_2_/FiO_2_ (mmHg)	178 (131–240)	200 (146–247)	165 (124–212)	0.588
Tidal volume (ml/kg PBW)	6.0 ± 2.2	6.1 ± 2.0	6.0 ± 2.4	0.914
PEEP (cm H_2_O)	12.0 ± 3.3	12.3 ± 3.2	11.7 ± 3.3	0.202
Peak inspiratory pressure (cm H_2_O)	31.7 ± 5.6	30.6 ± 5.1	32.8 ± 5.9	0.018
Mean airway pressure (cm H_2_O)	17.7 ± 4.0	17.4 ± 3.6	17.9 ± 4.3	0.406
Dynamic driving pressure (cm H_2_O)	19.8 ± 6.3	18.3 ± 6.0	21.1 ± 6.4	0.006
Dynamic compliance (ml/cm H_2_O)	19.2 ± 8.1	21.1 ± 7.7	17.4 ± 8.1	0.006
Duration of ECMO (days)	9.0 (4.8–14.6)	8.7 (5.0–13.0)	9.8 (4.7–16.1)	0.696
ECMO complications	43 (27.2%)	16 (22.5%)	27 (31.0%)	0.232

Data are presented as mean ± standard deviation, count or median (interquartile range)

*APACHE* Acute Physiology and Chronic Health Evaluation, *ARDS* acute respiratory distress syndrome, *ECMO* extracorporeal membrane oxygenation, *FiO*_*2*_ fraction of inspired oxygen, *PaCO*_*2*_ partial pressure of carbon dioxide in arterial blood, *PaO*_*2*_ partial pressure of oxygen in arterial blood, *PBW* predicted body weight, *PEEP* positive end-expiratory pressure, *SOFA* Sequential Organ Failure Assessment. Dynamic driving pressure: (peak inspiratory pressure—PEEP)

^a^Chronic heart disease included chronic heart failure, valvular heart disease, arrhythmia, hypertension and coronary artery disease

^b^Chronic lung disease included chronic obstructive pulmonary disease, interstitial lung disease, tuberculosis and bronchiectasis

^c^Chronic liver disease included chronic hepatitis and cirrhosis

^d^Chronic kidney disease included chronic renal insufficiency with creatinine level above 1.5 mg/dl

^e^Immunocompromised included hematological malignancies, solid tumors, sold organ transplantation, long-term steroid or immunosuppressant use and human immunodeficiency virus infection

### Mechanical ventilator settings after ECMO support

**Fig. 2:**
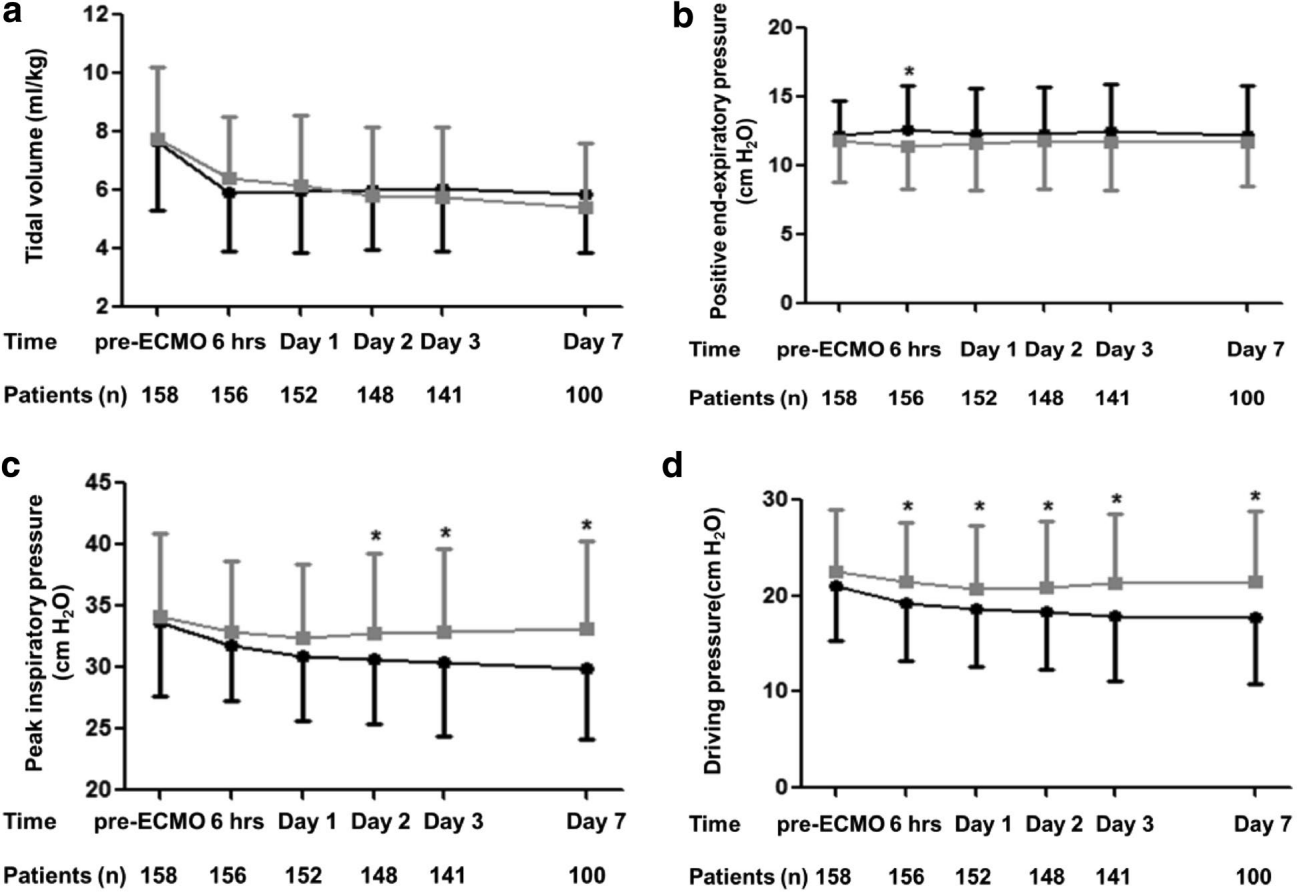
Serial changes in **a** tidal volume, **b** positive end-expiratory pressure (PEEP), **c** peak inspiratory pressure and **d** dynamic driving pressure before and after extracorporeal membrane oxygenation (ECMO). *Error bars* represent the mean ± standard error. *Dark line* denotes survivors and *gray line* denotes nonsurvivors. *A value of *p* less than 0.05 compared between survivors and nonsurvivors

Daily arterial blood gas, mechanical ventilation settings were recorded, and we analyzed the data at 6 h, day 1, 2, 3 and 7 after ECMO initiation. After ECMO support, tidal volume was reduced, but did not reveal significantly difference between survivors and nonsurvivors until day 7. After ECMO support 6 h, survivors had significant higher PEEP level than nonsurvivors (12.6 ± 3.3 vs. 11.4 ± 3.1 cm H_2_O, *p* = 0.02), but there was no difference until day 7. Both peak inspiratory pressure and dynamic driving pressure were decreased after ECMO initiation. Nonsurvivors had significantly higher peak inspiratory pressure after ECMO support day 2, day 3 and day 7 (32.8 ± 6.4 vs. 30.6 ± 5.2 cm H_2_O, *p* = 0.02; 32.9 ± 6.8 vs. 30.4 ± 6.0 cm H_2_O, *p* = 0.02; 33.1 ± 7.1 vs. 29.8 ± 5.7 cm H_2_O, *p* = 0.01). Nonsurvivors also had significantly higher dynamic driving pressure after ECMO support until day 7 (all *p* < 0.05) (Table 1; Fig. 2).

### Outcomes analysis

**Fig. 3:**
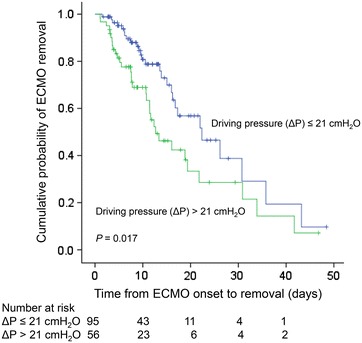
Time to ECMO removal analysis in patients with severe acute respiratory distress syndrome (ARDS) on extracorporeal membrane oxygenation (ECMO). *Blue line* denotes patients with mean dynamic driving pressure ≦21 cm H_2_O, and *green line* denotes patients with mean dynamic driving pressure >21 cm H_2_O from day 1 to 3 on ECMO (*p* = 0.017)

**Fig. 4:**
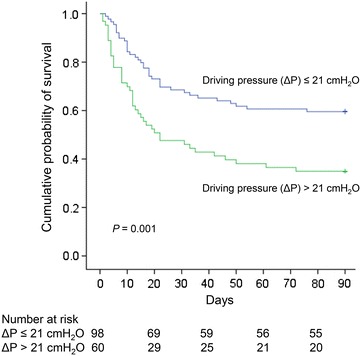
Kaplan–Meier survival curves in patients with severe acute respiratory distress syndrome (ARDS) on extracorporeal membrane oxygenation (ECMO). *Blue line* denotes patients with mean dynamic driving pressure ≦21 cm H_2_O, and *green line* denotes patients with mean dynamic driving pressure >21 cm H_2_O from day 1 to 3 on ECMO. The overall survival rate of patients with dynamic driving pressure ≦21 cm H_2_O was significantly higher than those with dynamic driving pressure >21 cm H_2_O (56.1 vs. 33.3%, *p* = 0.001)

Cox proportional hazards regression model was used to identify variables that have prognostic value for ICU mortality (Table 2). Immunocompromised status, APACHE II score, ARDS duration before ECMO and mean dynamic driving pressure from day 1 to 3 on ECMO remained independently associated with ICU mortality. Dynamic driving pressure with a cutoff point of 21 cm H_2_O exhibited the best Youden index, and mean dynamic driving pressure greater than 21 cm H_2_O from day 1 to 3 on ECMO was associated with higher mortality (HR 2.553; 95% CI 1.607–4.054; *p* < 0.001; data not shown). Peak inspiratory pressure and SOFA score were not retained in the final model due to highly correlated with dynamic driving pressure and APACHE II score, respectively. Time to ECMO removal analysis and a landmark analysis excluding 7 patients who died in the first 24 h after ECMO revealed that severe ARDS patients with mean dynamic driving pressure ≦21 cm H_2_O had significantly higher rate of ECMO removal than those with mean dynamic driving pressure >21 cm H_2_O from day 1 to 3 on ECMO (*p* = 0.017, log-rank test) (Fig. 3). The overall survival rate of severe ARDS patients with mean dynamic driving pressure ≦21 cm H_2_O was significantly higher than those with mean dynamic driving pressure >21 cm H_2_O from day 1 to 3 on ECMO (56.1 vs. 33.3%, *p* = 0.001, log-rank test) (Fig. 4).

**Table 2:** Cox proportional hazards regression model with ICU mortality as outcome

Factors	Hazard ratio (95% CI)	*p* value
Univariate analysis		
Age	1.011 (0.998–1.025)	0.108
Pulmonary contusion	0.417 (0.181–0.958)	0.039
Aspiration pneumonia	0.405 (0.128–1.285)	0.125
Diabetes mellitus	0.635 (0.373–1.083)	0.096
Chronic liver disease	1.611 (0.931–2.788)	0.088
Immunocompromised	1.731 (1.115–2.689)	0.015
APACHE II score	1.032 (1.004–1.062)	0.027
Lung injury score	0.596 (0.374–0.951)	0.030
ARDS duration before ECMO	1.002 (1.001–1.003)	0.001
Mean PEEP from day 1 to 3 on ECMO	0.942 (0.877–1.013)	0.106
Mean dynamic driving pressure from day 1 to 3 on ECMO	1.052 (1.015–1.090)	0.005
Mean dynamic compliance from day 1 to 3 on ECMO	0.971 (0.941–1.002)	0.069
Multivariate analysis		
Immunocompromised	1.957 (1.216–3.147)	0.006
APACHE II score	1.039 (1.005–1.073)	0.023
ARDS duration before ECMO	1.002 (1.000–1.003)	0.029
Mean dynamic driving pressure from day 1 to 3 on ECMO	1.070 (1.026–1.116)	0.002

*APACHE* Acute Physiology and Chronic Health Evaluation, *ARDS* acute respiratory distress syndrome, *CI* confidence interval, *ECMO* extracorporeal membrane oxygenation, *ICU* intensive care unit, *PEEP* positive end-expiratory pressure. Dynamic driving pressure: (peak inspiratory pressure—PEEP)

## Discussion

Our study analyzed the serial ventilator settings changes in severe ARDS patients after ECMO support and found that increased dynamic driving pressure during the first 3 days was independently associated with higher mortality. In addition, immunocompromised status, APACHE II score and the duration of ARDS before ECMO initiation were also significantly associated with survival.

Amato and colleagues analyzed nine randomized controlled trials in ARDS patients and concluded that driving pressure was most strong predictor of mortality [17]. Recent study also demonstrated decreased respiratory system and transpulmonary driving pressure were associated with improved 28-day mortality in ARDS patients [18]. A prospective multicenter study in 15 moderate ARDS patients with low-flow extracorporeal carbon dioxide removal (ECCO_2_R) demonstrated that driving pressure was significantly reduced during the first two days compared to baseline [19, 20]. However, the role of driving pressure on the severe ARDS patients requiring ECMO was uncertain, and a clinical review recommended that driving pressure is important determinant of outcome during ECMO [6]. The present study in severe ARDS patients receiving ECMO revealed that dynamic driving pressure from day 1 to 3 on ECMO was independently associated with mortality (Table 2).

Driving pressure was inversely proportional to compliance of respiratory system and had two common definitions: the difference between plateau pressure and PEEP and the difference between peak inspiratory pressure and PEEP [21]. There was no study compared different modes of ventilation during ECMO, and pressure-controlled mode appears to be advocated [12]. With pressure-controlled ventilation, pressure is maintained constant throughout inspiration, and flow decreases during inspiration and is often followed by a period of zero flow at end inspiration. Peak inspiratory pressure and peak alveolar pressure (plateau pressure) may be equal during no flow status [22]. Therefore, we used the difference between peak inspiratory pressure and PEEP as calculation of “dynamic” driving pressure. In fact, the most correct form can be obtained using transpulmonary driving pressure by esophageal manometry, but it is not easy to use in clinical practice. Reduction in dynamic driving pressure were found after ECMO initiation, and the values of survivors continued decreasing and exhibited significantly lower than nonsurvivors until day 7 (Fig. 2). Better lungs compliance and larger proportion of recovered functional lung size could have benefitted the survivors. Manipulation of driving pressure could be applied for ventilator management beside by adjusting the tidal volume and PEEP [18]. Although standardized ventilation protocol for ARDS patients before and after ECMO was followed, it remains unclear from our observational study to definitely conclude that driving pressure was causally related to outcome or simply another marker for ARDS severity and it needed further randomized controlled trials to confirm our findings.

Although ECMO facilitates the use of lung-protective ventilation, the optimal mechanical ventilation management is unknown [4, 6, 11–14]. The lowering levels of plateau pressure and tidal volume have been related to decreased mortality [16]. Therefore, an ultra-protective ventilation strategy with low tidal volume reduction (<4 ml/kg, PBW), airway pressure reduction and adequate PEEP was suggested to mitigate further VILI [4, 11–14]. Mechanical ventilation during ECMO may have an important impact on mortality. A cohort study of influenza A (H1N1)-induced ARDS patients receiving ECMO revealed that higher plateau pressure on the first day under ECMO was significantly associated with increased ICU mortality [23]. Another retrospective study demonstrated that higher PEEP levels during the first 3 days on ECMO were independently associated with lower ICU mortality [24]. A systemic review summarized ventilation practices in ARDS patients with ECMO, and mortality was lower among patients who had lower ventilation intensity following ECMO initiation [14]. Our present study found that pre-ECMO ventilator settings exhibited no significant difference. After ECMO initiation, tidal volume, peak inspiratory pressure and dynamic driving pressure were all decreased, while PEEP levels were relative maintained. Dynamic driving pressure during first 3 days of ECMO support was independently related to ICU mortality. Whether these mechanical settings affected the outcome was not well known, and more information will be obtained from an ongoing study in the future (SOLVE ARDS: Strategies for Optimal Lung Ventilation in ECMO for ARDS; clinicaltrials.gov identifier NCT01990456).

Several studies had investigated the predictors of mortality for severe ARDS patients treated with ECMO [10, 23–27]. Our study found that the duration of ARDS, APACHE II score and immunocompromised status before ECMO were independently associated with ICU mortality. The optimal timing for ECMO initiation had not been established, and mechanical ventilation may cause substantial VILI even under lung-protective strategy, which is worsened by delaying ECMO application for refractory hypoxemia [4, 13, 26–28]. Previous studies manifested duration of mechanical ventilation prior to ECMO support was correlated with mortality [10, 24–27]. Our study found that survivors had significantly shorter ARDS duration before ECMO. Several studies reported that degree of systemic organ failure was correlated with outcome for ARDS patients before ECMO initiation [6, 24–26, 29, 30], and we found that APACHE II score was significantly associated with ICU mortality. Furthermore, recent report included 2355 patients with severe ARDS receiving ECMO from multiple countries over a 13-year period concluded that immunocompromised status was independently associated with hospital survival [10]. Immunocompromised status was independently associated with long-term outcomes form severe ARDS patients with ECMO [25]. Our study also found that immunocompromised status was significantly related to ICU mortality.

There were several limitations of our study. First, this study is a retrospective analysis in one referral medical center, which may limit the generalization to other ICUs or hospitals. Besides, there might be residual and unmeasured confounding variables not included in our study and other biases during long period of study from 2006 to 2015 that could influence outcome. Second, APACHE II score was assessed only on the day of ICU admission and may not really reflect the dynamics of critical illness and treatment response. Serial evaluation of organ dysfunction during study period may be a better predictor of prognosis. Third, early application of prolonged prone position for severe ARDS patients as rescue therapy had survival benefit, but only a small number of our patients (*n* = 2) underwent prone position before ECMO. Finally, although ultra-protective ventilation strategy with ECMO based on a tidal volume reduction (<4 ml/kg, PBW), our study showed relatively higher tidal volume (around 6 ml/kg, PBW) after ECMO support.

## Conclusions

Our study found that immunocompromised status, APACHE II score and the duration of ARDS before ECMO initiation were significantly associated with ICU survival in severe ARDS patients with ECMO. Dynamic driving pressure during first 3 days of ECMO support was also independently related to ICU mortality. Further large multicenter, prospective randomized controlled trials are necessary to confirm the hypothesis that dynamic driving pressure could be a better predictor for survival in severe ARDS patients with ECMO support.

## Abbreviations

APACHE: Acute Physiology and Chronic Health Evaluation
ARDS: acute respiratory distress syndrome
CI: confidence interval
ECMO: extracorporeal membrane oxygenation
HR: hazard ratio
ICU: intensive care unit
PBW: predicted body weight
PEEP: positive end-expiratory pressure
SOFA: Sequential Organ Failure Assessment
VILI: ventilator-induced lung injury
